# Chemical Methods for Peptide and Protein Production

**DOI:** 10.3390/molecules18044373

**Published:** 2013-04-12

**Authors:** Saranya Chandrudu, Pavla Simerska, Istvan Toth

**Affiliations:** 1 School of Chemistry and Molecular Biosciences, The University of Queensland, St. Lucia, Qld 4072, Australia; E-Mails: s.chandrudu@uq.edu.au (S.C.); i.toth@uq.edu.au (I.T.); 2 School of Pharmacy, The University of Queensland, St. Lucia, Qld 4072, Australia

**Keywords:** peptide, protein, native chemical ligation, thioester, peptide synthesis

## Abstract

Since the invention of solid phase synthetic methods by Merrifield in 1963, the number of research groups focusing on peptide synthesis has grown exponentially. However, the original step-by-step synthesis had limitations: the purity of the final product decreased with the number of coupling steps. After the development of Boc and Fmoc protecting groups, novel amino acid protecting groups and new techniques were introduced to provide high quality and quantity peptide products. Fragment condensation was a popular method for peptide production in the 1980s, but unfortunately the rate of racemization and reaction difficulties proved less than ideal. Kent and co-workers revolutionized peptide coupling by introducing the chemoselective reaction of unprotected peptides, called native chemical ligation. Subsequently, research has focused on the development of novel ligating techniques including the famous click reaction, ligation of peptide hydrazides, and the recently reported α-ketoacid-hydroxylamine ligations with 5-oxaproline. Several companies have been formed all over the world to prepare high quality Good Manufacturing Practice peptide products on a multi-kilogram scale. This review describes the advances in peptide chemistry including the variety of synthetic peptide methods currently available and the broad application of peptides in medicinal chemistry.

## 1. The World of Peptides

**Scheme 1:**
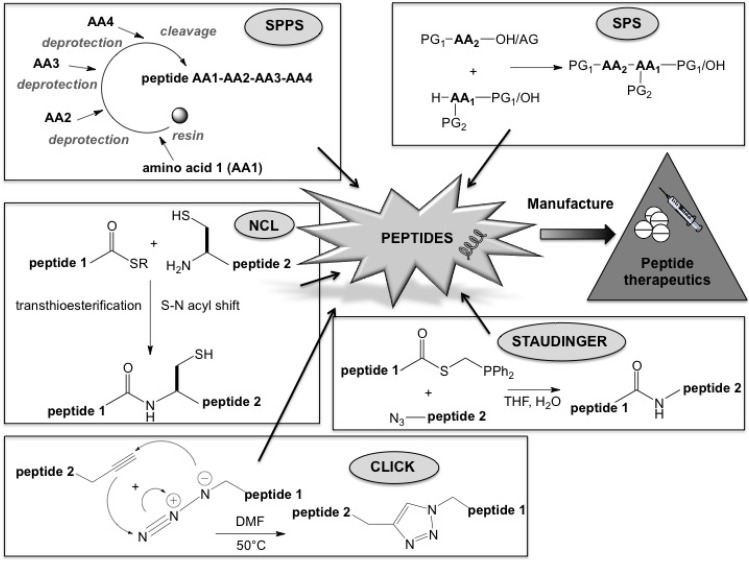
Schematic representation of peptide production.

Peptide synthetic techniques based on chemical methods have over 100 years of history (Table 1, Scheme 1). In 1881, Theodor Curtius synthesized the first N-protected dipeptide, benzoylglycylglycine, using the azide-coupling method, where the silver salt of glycine was treated with benzoylchloride. However, the first published synthetic dipeptide, glycylglycin, was synthesized by hydrolysis of the glycine diketopiperazine by Emil Fischer in 1901 and is considered the beginning of peptide chemistry [1,2]. Temporary amino-protecting groups had to be developed to overcome synthetic difficulties. The carbobenzoxy (Cbz) group was introduced in 1931 by Bergmann and Zerwas, followed by the *tert*-butyloxycarbonyl (Boc) group in 1957 by Carpino, McKay and Albertson [3,4,5]. Merrifield achieved a breakthrough discovery of solid phase peptide synthesis (SPPS) in 1963, when solid support was utilized for the synthesis of peptide sequences [3]. The major limitations of SPPS included incomplete coupling and deprotection reactions, accumulation of byproducts, and aggregation of growing peptides [6,7,8]. Synthesis of proteins by SPPS was also not feasible because the average length of a protein is approximately 250 amino acids [9]. To overcome the limitations of SPPS, new techniques for the synthesis of proteins have been developed; chemical ligation has been one of the most successful. Coupling two peptide fragments together, prior thiol capture strategy, was introduced by Kemp *et al.* [10]. Other ligation methods include native chemical ligation (NCL), expressed protein ligation (EPL), and Staudinger ligation. Additionally, the *O*-acyl isopeptide, also known as ‘click’ or ‘switch’ peptide, method is useful for the chemical assembly of highly aggregation-prone polypeptides [11]. 

**Table 1:** Important dates in peptide chemistry.

**Year**	**Discovery**
1901	First published synthesized dipeptide [2]
1957	Boc protecting group [12]
1963	SPPS discovery [3]
1967	HF cleavage [13]
1968	First automated solid phase synthesizer
1970	BHA resin [14]Fmoc protecting group [15]
1973	Wang resin [16]
1976	Preparative HPLC to purify peptides synthesized by SPPS
1977	Orthogonal protection [17]
1987	Rink resin [18]Sieber resin [19]
1992	Fast Boc protocol [20]
1994	NCL for protein and peptide synthesis [21]
1996	Pseudoprolines [22,23]

## 2. Chemical Methods for Peptide Synthesis

The two major chemical techniques for peptide production are SPPS and solution phase synthesis (SPS). 

Classical SPS is based on the coupling of single amino acids in solution. The fragment condensation method has been used for the synthesis of long peptides. In this case, short fragments of the required peptide are first synthesized, then coupled together to form a long peptide. The prime advantage of SPS for peptide synthesis is that the intermediate products can be deprotected and purified to give the final desired peptide in high purity [24,25]. Oxytocin (a neuromodulating nonapeptide and important hormone in sexual reproduction), porcine gastrin releasing peptide (a hormone stimulating secretion of gastric acid in the stomach), and human insulin (a 51 amino acid peptide hormone regulating carbohydrate metabolism in the body) are a few examples of peptide hormones that were synthesized by SPS [26,27]. Although SPS can be scaled up in an easy and inexpensive manner, the long reaction time remains a disadvantage. 

**Scheme 2:**
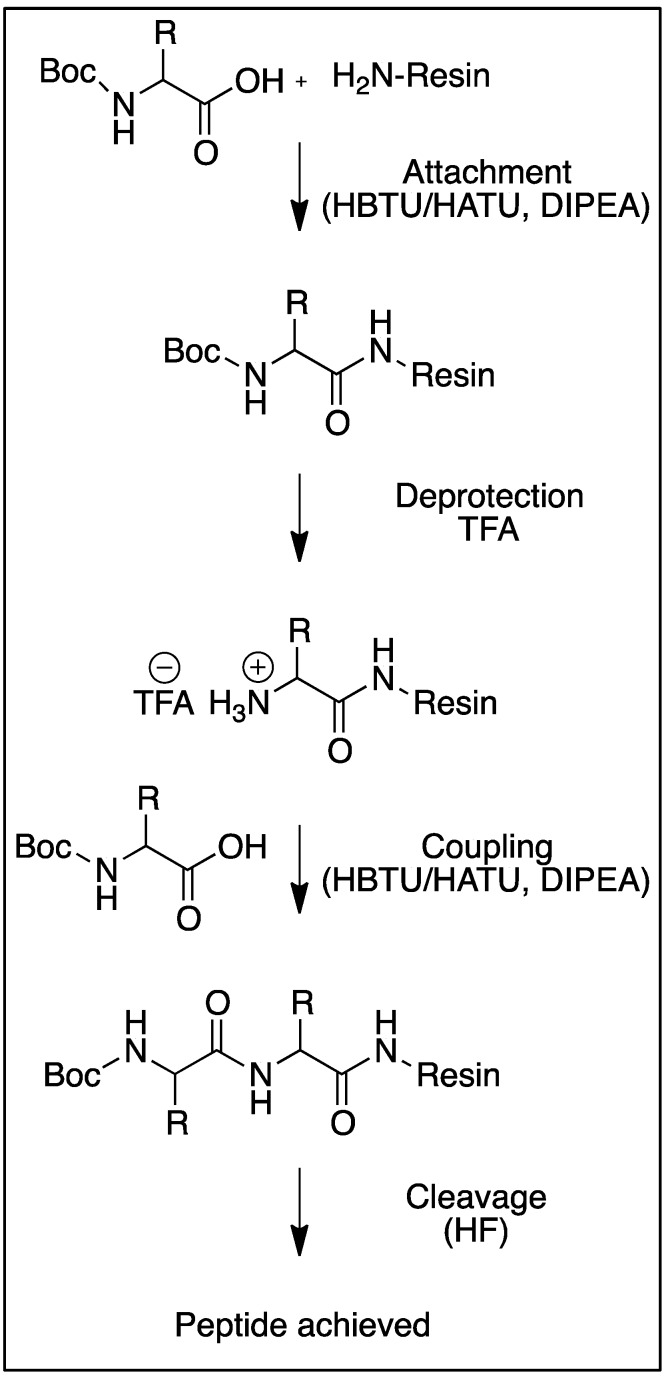
SPPS steps with Boc-chemistry.

During the SPPS method (Scheme 2), the resin is used as a support to which the growing peptide is anchored. First amino acid with temporary protecting groups on the reactive side chain and the alpha amino group (preventing polymerization) is attached to the resin via its C-terminus. After the addition of an amino acid, the protection group is removed and the resin washed prior to subsequent additions. The process is repeated until the sequence is completed, whereupon the required peptide is cleaved from the resin [3]. Boc and Fmoc protecting groups have often been used for side chain protection [4,15,28] and are removed by trifluoroacetic acid or 20% piperidine in dimethylformamide, respectively. Various resins have been used as a solid support in SPPS, for example: polystyrene, Merrifield, hydroxymethyl, phenylacetamidomethyl, Wang and 4-methylbenzhydrylamine resins [3,29,30]. 

SPPS synthesis was improved by the development of microwave-assisted SPPS especially when synthesizing long peptide sequences [20]. Microwave irradiation allowed for the synthesis of peptides in high yields and low degrees of racemization. Various peptides were synthesized in a shorter time when microwave-assisted SPPS was applied instead of the traditional SPPS. Another advantage of microwave-assisted SPPS use was the ability to control temperature and pressure while the synthesis was in progress. Generic time and temperature conditions for microwave-assisted SPPS using either Boc or Fmoc chemistry are listed below in Table 2. The limitations of this technique can be the cost of resin and equipment.

**Table 2:** *In situ* neutralization (optimized) protocol for microwave-SPPS [31,32].

**Synthetic Cycle**	**Reagents**	**Time & Conditions**
Deprotections	Trifluoroacetic acid (Boc chemistry)/20% piperidine in DMF (Fmoc chemistry)	1–5 min, 0 Watt, rt (Boc chemistry) or 70 °C (Fmoc chemistry)
Couplings	Amino acid, HBTU/HATU/HOBt/HOAt/DIC, DIPEA	5–15 min, 20 Watt,50–70 °C

## 3. Chemoselective Ligation Techniques

Chemical ligation was introduced as a convergent approach to the synthesis of long peptide or protein chains, where the smaller peptides were chemoselectively coupled in aqueous solution. Kemp developed the prior thiol capture method, which was used to couple two peptide fragments together [10,33]. The initial step, called thiol capture, involved the disulfide bond formation between the cysteine of the N-terminus and the thiol group of the C-terminus of the peptides. The acyl group was then transferred to form a native peptide bond [34]. 

**Scheme 3:**
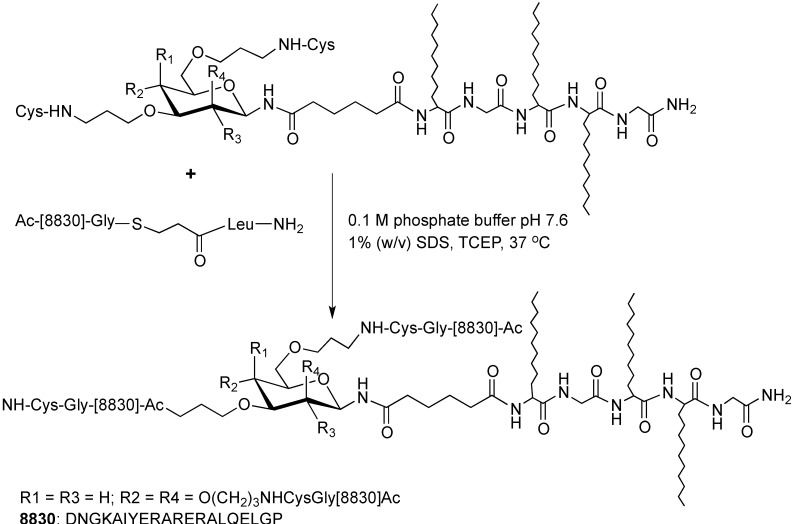
Application of NCL for development of a lipoglycopeptide vaccine [43].

Further optimization of chemical ligation led to the development of NCL, a technique where two or more unprotected peptide segments are assembled to form a large polypeptide. Since NCL was established in 1994 by Dawson, numerous proteins have been synthesized by this method, including human interleukin 8 [21]. For the ligation of two large polypeptide fragments, an unprotected peptide segment consisting of N-terminal cysteine was reacted with another unprotected peptide-α-thioester to generate a thioester-linked intermediate. This intermediate was transformed via an intramolecular acyl migration, which resulted in the formation of peptide bond. The reaction was carried out in buffered aqueous solutions of neutral pH as thioesters are not stable under basic conditions. The advantages of NCL include the high stability of the starting materials, high chemoselective nature, ligation of the unprotected segments, and well-established chemical methods for generating peptide thioesters [21,35]. NCL plays a significant role also in the synthesis of various proteins and more complex peptides. For example, the synthesis of a multivalent peptide-based nonsymmetrical dendrimer [36]. This dendrimer was prepared by NCL of two polylysine scaffolds, one bearing multiple copies of a peptide epitope and the second, a specific label. Another example of NCL application was the synthesis of high molecular weight collagen-like polymers, which would otherwise have been difficult due to their large size [37,38,39,40,41,42]. In this case, the N-terminal cysteine and *C*-terminal thioester peptides were prepared by SPPS, which were then polymerized through NCL under aqueous conditions and self-assembled into collagen-like polymers. Lipopeptide vaccine candidates against group A streptococcal infection were synthesized by NCL using similar principles (Scheme 3) [43]. 

Peptide-α-thioesters are the main building blocks for peptide synthesis by NCL and Boc-SPPS chemistry is one of the possible strategies that can be used for their synthesis. However, certain limitations like harsh cleavage conditions of the peptide product from the resin can limit its use. An alternative method would be the use of mild reagents during Fmoc chemistry [44]. It was found that a 0.88:1 ratio of diazabicycloudecene/*N*-hydroxybenzotriazole did not disrupt the thioester linkages while removing Fmoc group [45]. 1-Methylpyrrolidine, hexamethyleneimine and *N*-hydroxybenzotriazole in a 1:1 mixture of l-methyl-2-pyrrolidinone and dimethyl sulfoxide can be also used as the deprotecting reagent [45]. In another method for the synthesis of peptide-α-thioesters, the peptide was assembled on a 3-carboxypropanesulfonamide linker through Fmoc SPPS [44], which involved an intramolecular N/S acyl shift on a sulfonamide safety catch linker [46]. An alternative strategy was introduced by Alsina *et al.*, and used the backbone amide linker for the synthesis of C-terminal modified peptides [47]. The C-terminus was first orthogonally protected before anchoring the penultimate residue of the target peptide to the resin, followed by peptide elongation from C to N terminus. After selective orthogonal deprotection, the C-terminus was modified with a chosen moiety and the product cleaved from the resin.

NCL is a useful strategy to overcome some of the size limitations of SPPS. Multiple ligation has been used to synthesize many problematic proteins, for example the synthesis of snow flea antifreeze protein, an 81 amino acid sequence that was difficult to synthesis by other means [48,49]. The resin, protecting group, and linker play all an important role in the ligation strategy. In order to increase the effectiveness of multiple segment ligation, another ligation technique called solid phase ligation was introduced [50]. In kinetically controlled NCL, the dual reactivity of a bifunctional Cys-peptide-1-thioester needs to be controlled when reacted with a peptide-2-thioester under NCL conditions to yield only a single product. This was achieved by employing the more reactive peptide-α-thiophenylester with a Cys-peptide-α-thioalkylester [51]. Human lysozyme (a 130-amino acid sequence) was one of the proteins synthesized using kinetically controlled NCL [51]. Levacher *et al.* designed a novel ligation technique for peptide bond formation using amine capture strategy and peptide-α-thioester quinolinium salt [52].

Recombinant proteins could not be converted into protein thioesters. Therefore, a new method, known as expressed protein ligation (EPL) was introduced for the semi-synthesis of proteins. This method was based on the naturally occurring protein splicing process [53]. In order to form a cysteine bound thioester, the cysteine residue at the *N*-terminus underwent an N-S shift. Following this step, the thioester was transferred to the cysteine residue, an intramolecular rearrangement with an asparagine residue occurred at the C-terminus [54,55,56]. The EPL strategy was used to prepare the *Consensus* tetratricopeptide repeat [57].

Staudinger ligation, which was developed by Raines and Bertozzi, is another alternative to NCL [58]. This method was based on the Staudinger reaction, where phosphane reacted with an azide to produce iminophosphorane. The iminophosphorane underwent an acyl shift that produced amidophosphonium salt. The amide product and phosphine oxide were formed after amidophosphonium salt hydrolysis. The Staudinger ligation technique has been employed for protein site-selective modification and peptide and protein immobilization [58,59,60]. This strategy has been used also to incorporate azobenzene switches into a target system, which bear the functionalities of an azide [61].

Click chemistry was described by Sharples in 2001 as a copper(I)-catalyzed 1,3-dipolar cycloaddition of alkynes to azides to form 1,2,3-triazoles. Since then, this method has been used in organic chemistry, particularly for drug discovery and development [62,63]. Studies showed that the solubility of peptides, especially that of long, easily aggregated sequences [64], was increased by the presence of an *O*-acyl instead of an *N*-acyl moiety [65]. For example, byproduct formation and solubility problems prevailed when a pentapeptide sequence was synthesized by the traditional method, but these issues were not encountered when click chemistry was used [66]. 

Another method for protein synthesis is the ligation of peptide hydrazides. This new ligation method does not require the use of protecting groups, which makes it attractive for modern chemical synthesis of proteins. Two unprotected model peptides were ligated in a one-pot reaction that included two steps. Firstly, two peptides were mixed with NaNO_2_ (an oxidant) under acidic condition to produce a peptide azide. Thiols were added, and the pH adjusted, resulting in the formation of the required product [67,68]. 

α-Ketoacid-Hydroxylamine ligation with the help of 5-oxaproline is another technique for chemical synthesis of proteins. For example this ligation method was used for the synthesis of two proteins, both from *Mycobacterium*. The first protein synthesized by this method was prokaryotic-ubiquitin-like protein and the second one was cold shock protein A [69,70,71,72].

## 4. Application of Peptides and Proteins in Medicinal Chemistry

Peptides are usually selective and efficacious, acting on their targets in low concentrations, thus are one of the best candidates for drug development and delivery. The Food and Drug Administration (FDA) has approved many peptide and peptide-based drugs for use as therapeutics [73,74,75,76]. Currently, there are more than 60 peptide drugs on the market and over 500 peptides are in various stages of preclinical and clinical development [77]. Peptide drugs have applications in the medical and pharmaceutical industry, especially for treatment of cancer (18%), and metabolic disorders (17%, including diabetes, obesity, osteoporosis), and other medical conditions such as allergy, immunological disorders, and cardiovascular disease [78]. In cancer research, Wilm’s tumour-1 peptide in dendritic cell-based vaccines was found to positively impact the survival of patients with non-small cell lung cancers [79]. Another possible therapeutic target, insulin B chain peptide recognized by a specific T cell receptor, was found to be responsible for initiation of diabetes [80]. B-Type natriuretic peptide produced by cardiomycetes was used clinically and represents an efficacious therapeutic strategy to treat human heart failure conditions [81]. Short peptides recently reported by Dawgul *et al.* were found to be active against *Staphylococcus aureus* infections thus could be used to treat staphylococcal skin disease [82]. 

**Scheme 4:**
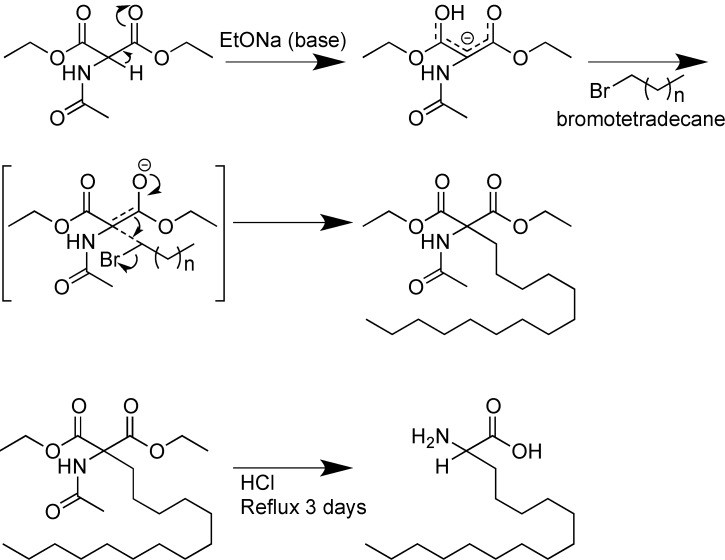
Lipoamino acid synthesis (n = a number of carbon atoms in the LAA’s side chain).

Toth *et al.* observed that incorporating α-amino acids with long alkyl chains, lipoamino acids (LAAs), into a therapeutic peptide sequence influenced its particle size [83], and more importantly increased the immunogenicity of peptides [84]. LAAs consisting of various alkyl chain length (e.g., the most commonly used carbon atom chain n = 9, C12, and n = 13, C16) were synthesized as shown in Scheme 4 and protected with either Boc- [84,85] or 2-acetyldimedone (Dde) [86] protecting groups depending on the chemistry used (Boc or Fmoc, respectively). 

Various modifications (e.g., lipidation, glycosylation) of therapeutic peptides [87] as well as numerous delivery systems have been studied for application of peptides in drug and vaccine delivery [88]. For example, opioid peptide endomorphin-1 modified with a lipid moiety demonstrated systemic activity against neuropathic pain [89]. Immunogenic peptides have been used for the development of group A streptococcal vaccine candidates using polylysine [90] or carbohydrate cores (Scheme 3) [91,92]. Standard and/or microwave-assisted techniques were applied to their synthesis [43,91]. These self-adjuvanting vaccine candidates were immunogenic when administered subcutaneously [93] and intranasaly [94] to mice. Another example is the B-cell peptide epitope from the apical enzyme in the hemoglobin digestion cascade, which was incorporated in vaccine candidates to target the major human hookworm [95]. The candidates were synthesized by standard SPPS and induced a strong IgG response in mice [95]. 

Proteins are another clinically and commercially important class of therapeutics. Streptokinase is a protein drug, which is nowadays available on the market for treatment of heart conditions, thrombosis, and embolism. Mutations of an adipocyte-specific secreted protein leptin were shown to decrease appetite and increase the metabolic rate in humans. This genetic evidence of leptin being an important regulator of energy balance could be a way to treat obesity [96]. Capsid proteins from four human papillomavirus strains were successfully utilized for vaccine development and marketed as prevention against cervical cancer [97].

## 5. Peptide Manufacture

The growth of peptides as therapeutics is progressively gaining the attention of drug companies. The advantages of peptides as medication include their specificity, efficacy, activity and low toxicity. Companies have been formed all over the world to provide high quality Good Manufacturing Practice peptide products. These include Bachem and Lonza (Switzerland), Biomatik (Canada), GenScript and AmbioPharm (USA), Polypeptide Laboratories (Europe, USA.) and Shym-Pharma (China) that produce peptides on a multi-kilogram scale. To date, several peptide therapeutics have reached global sales over US$1 billion, for example glatiramer acetate (Copaxone^®^, Teva Pharmaceutical), leuprolide acetate (Lupron^®^, Abbott), goserelin acetate (Zoladex, Astra Zeneca), and octreotide acetate (Sandostatin^®^, Novartis) (Table 3).

Chemical synthesis, especially SPPS based on Fmoc chemistry, is nowadays the most popular choice of manufacturing procedure for peptides. This is because the solid phase manufacturing cost decreased (due to the lower cost of raw materials, and economy of scale), and the technical improvements in chromatographic equipment and media. SPPS methods are faster, more flexible (in design of analogs) and less expensive (require less process development, use generic chemical and purification processes) for peptide manufacturing at up to a multi-100-kg scale than the use of recombinant technology. However, there are peptide therapeutics (human glucagon, salmon calcitonin, *etc.*), which are manufactured using recombinant technology and it is expected that the application of recombinant technology for peptide production will increase mainly due to its advantageous possibility for scale up. Some peptides, like an HIV fusion inhibitor (36 amino acids; fuzeon) or a direct thrombin inhibitor (20 amino acids; bivalirudin), are routinely manufactured on a large scale that exceeds 100 kg per year. To further improve cell penetration, stability, specificity and targeting of peptides, the hydrocarbon stapling technique was developed by Verdine *et al*. [98,99,100]. The bioactive alpha helical fold was introduced by the site-specific addition of a chemical brace. This method allowed the formation of stable alpha helical peptide structures important in many biological pathways, and has been used in linear, cyclic, and nanoparticle-based forms of peptides [101,102]. Since then, several companies (e.g., AnaSpec, Aileron Therapeutics) have applied this technique to enhance the pharmacological performance of therapeutic peptides.

**Table 3:** Examples of peptide based drugs available on the market.

**Generic Name**	**Trade Name**	**Disease Target**	**Company**
**Teriparatide**	Forteo	Osteoporosis	Eli Lilly & Co.
**Exenatide**	Byetta	Type 2 diabetes	Amylin/Lilly
**Enfuvirtide**	Fuzeon	HIV	Roche/Trimeris
**Degarelix**	Firmagon	Prostate cancer	Ferring
**Mifamurtide**	Mepact	Bone cancer	Takeda
**Nesiritide**	Natrecor	Heart failure	Johnson & Johnson
**Goserelin**	Zoladex	Breast and Prostate cancer	AstraZeneca
**Glatiramer**	Copaxone	Multiple sclerosis	Teva Pharmaceuticals
**Octreotide** **Lanreotide**	Sandostatin Somatuline, Angiopeptin	Neuroendocrine tumors	Novartis Pharmaceuticals Ipsen
**Icatibant**	Firazyr	Hereditary angioedema	Jerini
**Ziconotide**	Prialt	Pain	Elan, Azur Pharma
**Pramlintide**	Symlin	Diabetes	Amylin
**Romiplostim**	Nplate	Idiopathic thrombocytopenic purpura	Amgen

## 6. Conclusions

The field of peptide science is steadily growing and this tremendous progression shows the significance of peptides and proteins as therapeutics. All techniques described herein can be tailored to prepare a variety of peptides and proteins. Several peptide drugs have been already approved by FDA and have reached the market, which demonstrates the potential of peptides to be used as effective drugs. The breadth of opportunity offered by peptide therapeutics clearly demonstrates the present and future potential of the peptide chemistry field. 
